# The role of microRNA‐93 regulating angiopoietin2 in the formation of malignant pleural effusion

**DOI:** 10.1002/cam4.1000

**Published:** 2017-04-11

**Authors:** Qian Qian, Wenkui Sun, Wen Zhu, Yanan Liu, Ai Ge, Yuan Ma, Yu Zhang, Xiaoning Zeng, Mao Huang

**Affiliations:** ^1^ Department of Respiratory & Critical Care Medicinethe First Affiliated Hospital of Nanjing Medical University300# Guangzhou RoadNanjingJiangsu210029China; ^2^ Respiratory MedicineNanjing Chest HospitalMedicine School of Southeast University215# Guangzhou RoadNanjingJiangsu210029China

**Keywords:** Angiogenesis, angiopoietin2, lung cancer, microRNA, pleural effusion

## Abstract

The biological roles of miRNAs in the development of malignant pleural effusion (MPE) are unclear. In this study, the miRNA microarray analysis was performed in two different prognosis groups of lung adenocarcinoma patients. Expression profiles of miRNAs in MPEs were identified. With the help of quantification PCR, we confirmed the expression differences of miRNAs and further analyzed their biological functions and relative target genes in vitro. The target gene of miR‐93 was estimated by online database, and also, the protein was tested. The target gene and the binding sites of specific miRNA were estimated by online database. The combining capacity of binding sites was verified by luciferase reporter gene assay, and the target gene protein was tested by western blot. We detected 107 miRNAs with expression differences (*n* = 10) and confirmed significant expression differences in miR‐93 and miR‐146a in two groups of patients (*n* = 84). By manipulating miR‐93 expression of human lymphatic endothelial cells (HLEC) and human umbilical vein endothelial cells (HUVEC), we discovered that high expression of miR‐93 inhibited migration, proliferation, and angiogenesis. And also, miR‐93 increased not only apoptosis, but also G1 phase cell block. By using luciferase reporter gene assay and western blot, we confirmed that angiopoietin2 (Ang2) was the target of miR‐93. The data showed that miR‐93 has an inhibiting effect on pleural effusion. By targeting Ang2, miR‐93 regulates angiogenesis and lymphangiogenesis and plays a role in pathogenetic mechanism of MPE. MiR‐93/Ang2 may shed light on potential new targets in cancer treatment.

## Introduction

Malignant pleural effusion (MPE) is the sign of an advanced or generalized tumor stage, and it complicates the course of various malignancies, such as lung and breast adenocarcinoma. MPE has been found in 15% of newly diagnosed patients with non‐small‐cell lung cancer (NSCLC), which is one of the signs of poor prognosis in patients with lung cancer 1, 2. Most patients can only be offered with symptomatic and palliative treatment to alleviate breathlessness 3. And the currently used standard palliative methods (pleural puncture, long‐term placement of drainage tube and pleural fixation) are suboptimal 4.

At present, the exact pathogenetic mechanisms of exudative MPE are unclear. The increase in the permeability of microvasculature generally attributes to the leakage of MPE. Pleural fluid accumulates when production outweighs removal, and the latter mainly occurs via pleuropulmonary lymphatics. Angiogenesis and lymphangiogenesis in metastatic tumor of pleura contribute to the development of MPE 5.

MiRNAs are endogenous noncoding RNAs of 20–22 nucleotides regulating gene expression at the post‐transcriptional level. It is shown that many kinds of miRNAs are located in cancer‐associated genomic region, acting as oncogenes or tumor suppressors 6, 7, 8, 9. Notably, many studies focus on the potential use of miRNAs in MPE as biomarkers for diagnosis, progression, and prognosis of tumors. For example, the detection of miRNA‐21 and miRNA‐155 expressions in pleural effusion shows high specificity and sensitivity in the diagnosis of malignant pleural effusion 10, 11. More efforts are required to clarify the role and mechanism of miRNAs in MPE generation.

In this study, we identified the expression profiles of MPE miRNAs in a cohort of lung adenocarcinoma patients. Integrated analysis was performed on lung adenocarcinoma patients with MPE through prognostic approaches. We further investigated both miR‐93 and angiopoietin2 (Ang2) in angiogenesis and lymphangiogenesis of endothelial cells. Addressing these questions will help us understand the pathogenesis of MPE.

## Materials and Methods

### Patients and sample collection

MPE samples were obtained from lung adenocarcinoma patients who were admitted to the Nanjing Chest Hospital from September 2010 to December 2011. Lung adenocarcinoma patients with the following characteristics were enrolled: (1) diagnosis with cytologic or histologic confirmation in MPE, pleura; (2) new diagnosis and no treatment for lung cancer (patients with pleural mesothelioma and metastatic carcinoma of other tumors were excluded.). Pleural effusion was obtained through thoracentesis or thoracic drainage guided by B‐ultrasound. Then, the samples were centrifuged at 1000 g for 10 min and the supernatant was obtained and stored at −80°C for further analysis. The study was approved by the Medical Ethical Committee of the Nanjing Chest Hospital (Nanjing, China) and the written informed consent was obtained from all enrolled patients.

### RNA extraction

According to the manufacturer's instructions, we used the miRcute miRNA isolation kit (Tiangen, Beijing, China) to extract the enriched miRNA from fresh‐frozen samples and cells. Total RNA was extracted from cells by using the TRIzol reagent (Invitrogen, Carlsbad, CA, USA) according to the manufacturer's protocol.

### MicroRNA microarray and data analysis

Total RNA was reversely transcribed into the first strand of cDNA by using the TaqMan MicroRNA Reverse Transcription Kit and the Megaplex RT Primers, Human Pool A+B (Applied Biosystems, FosterCity, CA). MicroRNA expression profiling was performed by using the TaqMan Universal PCR Master Mix. These tests were carried out by using 7900HT fast Real‐Time PCR System (Applied Biosystems) according to the manufacturer's protocol. In this study, we compared miRNAs expression profiles between two groups of patients with different prognosis. The differentially expressed miRNAs were chosen according to the *P*‐value threshold and fold changes from significant analysis and FDR analysis 12. Ten of these miRNAs were chosen for further study.

### Quantitative real‐time PCR verification of miRNAs

Complementary DNA (cDNA) was synthesized from RNA with miRNA‐specific stem‐loop reverse transcription (RT) primers by using the miRcute miRNA First‐Strand cDNA Synthesis Kit (Tiangen, Beijing, China) according to the manufacturer's protocol. Real‐time PCR was performed by using an ABI 7500 system (ABI, Foster City, CA). MiRcute miRNA qPCR Detection Kit (SYBR Green) (Tiangen, Beijing, China) was used to confirm the miRNA expression. And U6 snRNA (small nuclear RNA) was used as an internal control. All miRNAs were measured by using the equation $2^{-\Delta C_{t}}\left(\Delta C_{t}=C_{mi\mathrm{RNA}}-{\text{C}}_{tU6}\right)$ 13.

#### Cell lines

Epithelial cell lines (human lymphatic endothelial cells [HLEC] and human umbilical vein endothelial cells [HUVEC]) and human embryonic kidney cell HEK‐293 were used in this study. HLEC, HUVEC, and HEK‐293 were obtained from NeuronBiotech (Shanghai, China). HLEC and HEK‐293 were cultured in complete DMEM high glucose medium (Gibco, Gaithersburg, BRL, USA) supplemented with 10% FBS (fetal bovine serum)(Hyclone, Logan, UT, USA) and 1% penicillin and streptomycin sulfate (Beyotime Biotechnology Company, Jiangsu, China). HUVEC was cultured in RPMI1640 medium (Gibco) supplemented with 10% FBS and 1% penicillin and streptomycin sulfate. Cells were incubated at 37°C with 5% CO^2^ and the medium was changed every 2 or 3 days.

### Cell migration and proliferation assay

Transwell cell migration assay and Cell Counting Kit‐WST8 (CCK8) were carried out to investigate the impact of miR‐93 on the migratory and proliferation ability of HUVEC and HLEC. To detect the migration, cells were seeded on top of the transwell membrane at 3 × 10^4^ cells/well in serum‐free DMEM for HLEC, and RPMI1640 for HUVEC 48 h after the transfection. The lower chamber containing 10% FBS was used as the chemoattractant. A duration of 24 h later, cells were fixed with 95% alcohol and stained with 0.05% crystal violet for 30 min. Cells in five random fields of each membrane were counted. Cell proliferation was investigated by using the WST‐1 assays. Cells were placed in 96‐well plates 48 h after the transfection, and the serum was starved for 24 h before grown in 10% FBS medium. Proliferation was ceased, respectively, 24 h, 48 h, 72 h, and 96 h later. The proliferative effect of miR‐93 was assessed by Cell Count Kit‐8 (Beyotime Biotechnology Company, Jiangsu, China). To measure the colony‐forming activity, HLEM and HUVEM were counted and seeded into plates, respectively, with 400, 800, 1200, 1600 cells per plate. The culture medium was replaced every 3 days. Ten days after seeding, the number of colonies containing more than 50 cells was counted.

### Matrigel plug angiogenesis assay

Matrigel assay was performed in 6‐well plate, which was previously coated with matrigel basement membrane (50 *μ*L/cm^2^, 10 mg/mL) (BDBiosciences, Vienna, Austria). A duration of 24 h after the transfection, the concentration of cells in each group was adjusted to 2 × 10^5^ cells/mL. And then, 50 *μ*L cell suspension was inoculated in each well of the plate. The assay plate was incubated for 48 h at 37°C, 5% CO^2^ atmosphere. In each situation, pictures were taken by using phase contrast microscopy, and the mean tube length was calculated. Furthermore, branch points were counted to quantify angiogenic capacity of cells by using MetaMorph.

### Prediction of miRNA target genes

The miRNA analysis web (http://www.microrna.org/) was used to nominate the candidate miRNAs. MiRNAs targeting at angiopoietin2 (Ang2) were predicted by the analysis web. This web‐based tool is integrated with seven most often used target gene prediction algorithms: DIANA, miRanda, PITA, rna22, miRBridge, PicTar, and TargetScan.

### Plasmid construction and dual luciferase reporter assay

Plasmids of pGL3‐Ang2, pGL3‐mu‐Ang2, pRL8‐SV40, and pGL3‐Basic vector were constructed and used. The fragments of the Ang2 3′‐untranslated region (UTR region) (1933–1956 bp) were prepared for luciferase constructs, which contain potential target sites of miR‐93(1928–2511 bp). The fragments were amplified by PCR with the following primers: for pGL3‐Ang2 sense, 5′‐CCGAGCTCTTTCCACCAAGGTGCACTTTTCCAA‐3′; and anti‐sense, 5′‐CCCTCGAGAGTTGAACTGTGCTCCCTGATC‐3′; and for mutant plasmid of pGL3‐mu‐Ang2 sense, 5′‐CCGAGCTCTTTCCACCAAGCTCCTCATTTCCAA‐3′; and anti‐sense, 5′‐CCCTCGAGAGTTGAACTGTGCTCCCTGATC‐3′. PGL3‐Ang2 is constructed with the 1933‐1956 binding sites, which are the miR‐93 binding sites in the 3′UTR of Ang2. In addition, the plasmid containing mutant Ang2 3′‐UTR binding sites was generated. For the reporter assay, luciferase constructs (11 ng) were transfected into the HEK‐293T cells by using Lipofectamin 2000 (Invitrogen, Carlsbad, CA, USA) with 20 nmol/L of miRNA mimics. A duration of 48 h after the transfection, cells were harvested and the luciferase intensity was measured by using a Dual Luciferase Reporter Gene Assay kit (Promega, Madison, WI) according to the manufacturer's instructions. To obtain relative luciferase activity, firefly luminescence was normalized by the renilla luminescence.

### Statistical analysis

The statistical differences observed were evaluated by paired Student's *t*‐test or one‐way ANOVA. All analyses were performed by using SPSS13. All reported *P* values were two‐sided, with *P *<* *0.05 considered statistically significant.

## Results

### General information

According to the inclusion and exclusion criteria in this study, 94 cases of lung adenocarcinoma with MPE were included. Ten of them were enrolled in miRNA microarray. According to PFS, these 10 cases were divided into two groups: the good prognosis group (PFS ≤ 2M, five cases) and the poor prognosis group (PFS ≥ 5M, five cases). The enlargement of PFS may enhance the detection efficacy of miRNA microarray. After the microarray analysis, further validation was performed on the left 84 cases, including 43 cases with good prognosis (PFS ≥ 4M) and 41 cases with poor prognosis (PFS < 4M). The median PFS of these 84 cases is 4 months. Both groups suffered from lung adenocarcinoma, and there was no significant difference in their disease stage, age, smoking status, and gender (Table 1) (*P *>* *0.05).

**Table 1:** Characteristics of patients in the group

Characteristics	Microarray research		Verification research	
	Good prognosis(5)	Poor prognosis(5)	Good prognosis(43)	Poor prognosis(41)
Gender (*n*)				
Male	3	5	29	25
Female	2	0	14	16
Age				
<60 years old	3	1	19	13
≥60 years old	2	4	24	28
ECOG score				
0	2	2	19	17
1	2	3	18	15
2	1	0	6	9
Smoking status (*n*)				
Smoker	2	3	31	26
Nonsmoker	3	2	12	15

### The miRNA‐microarray analysis of MPE

**Figure 1:**
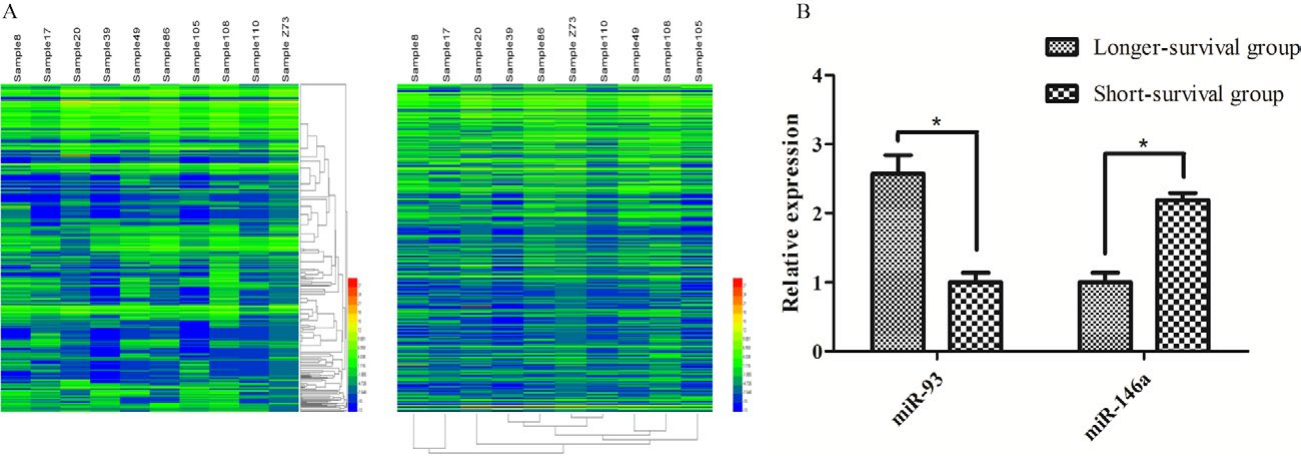
MPE miRNAs of lung adenocarcinoma patients. (A) The microarray detection results of miRNAs(Cluster Analysis). (B) The expression of miR‐93 and miR‐146a in pleural effusion through real‐time quantification RT‐PCR. *P <* *0.05. MPE, malignant pleural effusion.

Ten cases of MPE were investigated via miRNA microarray (Applied Biosystems). Five cases had good prognosis (PFS ≥ 5M), and others had poor prognosis (PFS ≤ 2M) (Fig. 1). After raw data normalization, 107 miRNAs were found to be differentially expressed in two groups, and 33 of them have fold‐changes ≥2.0 (File S1). By carrying out bioinformatic analysis, 10 miRNAs were chosen to be verified in 84 cases through real‐time quantification RT‐PCR (reverse transcription PCR). We discovered that the expression differences in miR‐93 and miR‐146a showed statistical significant difference in two groups (Fig. 1).

### Manipulation of miR‐93 in cell lines and PCR verification experiment

**Figure 2:**
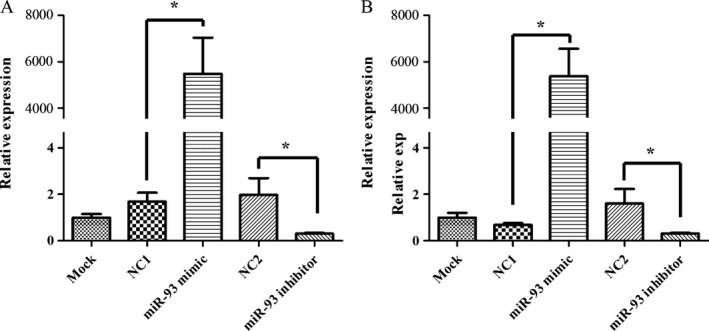
PCR detection of miR‐93 expression changes in cell lines after transient transfection. Expression levels of miR‐93 in two cell lines were indicated. Cells were transfected with either 20 *μ*mol/L of miR‐93 mimic, miR‐93 inhibitor, and NC‐miRNA. A duration of 48 h after transfection, the expression of miR‐93 was detected and normalized to internal GAPDH and U6 controls, respectively. (A) HLEC. (B) HUVEC. *P <* *0.05. HUVEC, human umbilical vein endothelial cells.

Transient transfection was carried out by using lipofectin technique. MiR‐93 mimic, miR‐93 inhibitor, and negative control‐miRNA (NC‐miRNA) were transfected into HUVEC and HLEC. A duration of 48 h after the transfection, cellular RNA was extracted, and then, the expression of miR‐93 was detected by using quantification RT‐PCR. Relative expression was calculated and then compared with U6 (Fig. 2). The results showed that when miR‐93 mimic was transfected with HUVEC, its expression was 5185.72 times higher than the control group(*P *<* *0.05); when miR‐93 inhibitor was transfected with HUVEC, its expression was 0.32 times lower than the control group(*P *<* *0.05); when miR‐93 mimic was transfected with HLEC, its expression was 5487.9 times higher than the control group(*P *<* *0.05); when miR‐93 inhibitor was transfected with HLEC, its expression was 0.31 times lower than the control group (*P *<* *0.05).

### MiR‐93 regulates endothelial cells motility

**Figure 3:**
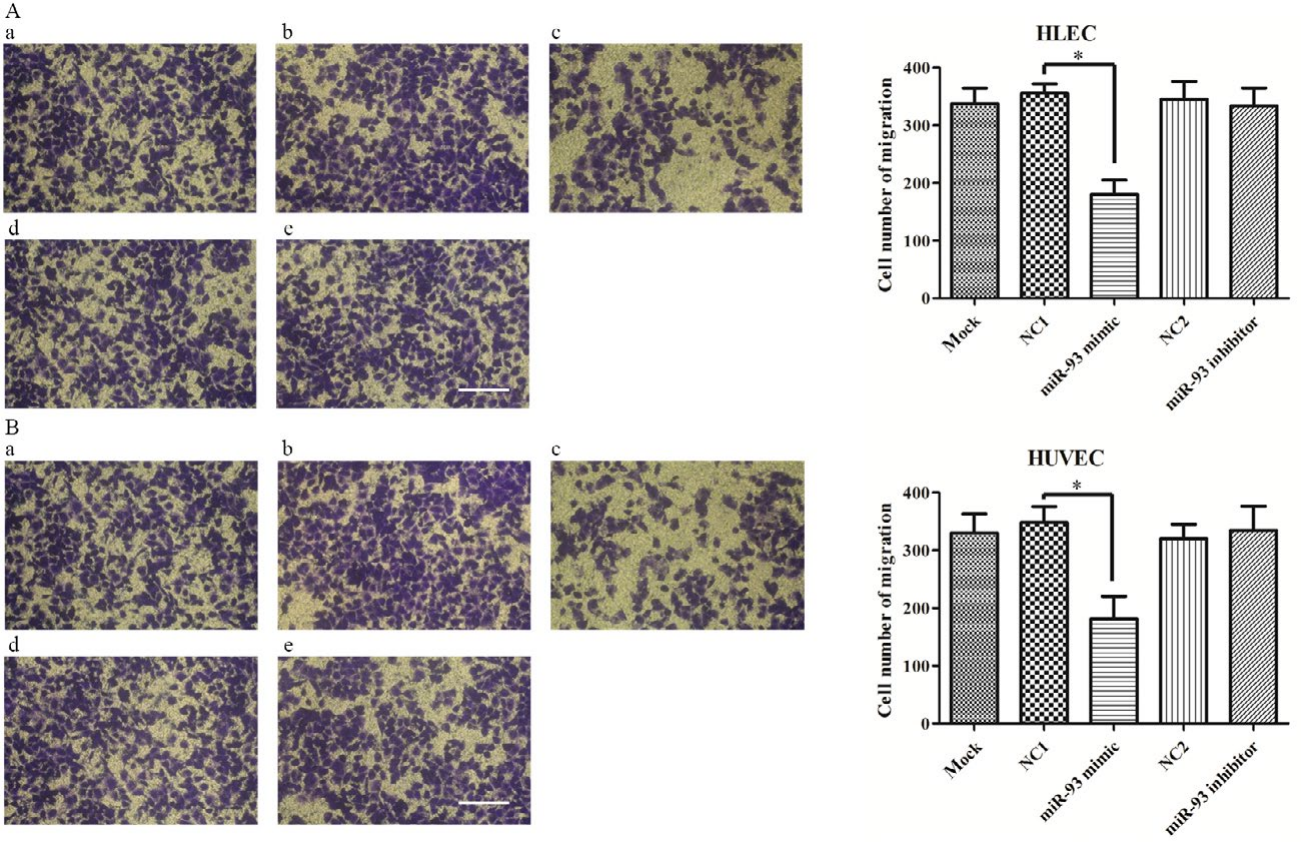
Effect of miR‐93 on cell migration in HLEC and HUVEC. A duration of 24 h later, the cells that had migrated through the membrane were fixed with 95% alcohol and stained with crystal violet. The number of migrated cells was quantified by counting five independent symmetrical visual fields under the microscope (×100). The number of HLEC and HUVEC transfected with miR‐93 mimic passing through the membrane was significantly lower than that of HLEC and HUVEC transfected with negative controls. (A) HLEC. (B) HUVEC. (a) blank control. (b) and (d) negative control (NC‐miRNA). (c) miR93 mimic. (e) miR‐93 inhibitor. Scare bar = 100 *μ*m. *P <* *0.05. HUVEC, human umbilical vein endothelial cells.

**Figure 4:**
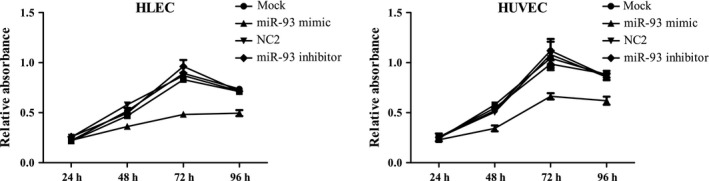
Effect of miR‐93 on cell proliferation in HLEC and HUVEC with WST‐1 assay. HLEC and HUVEC proliferation were tested 24 h, 48 h, 72 h, and 96 h later. The proliferation ability of HLEC and HUVEC decreased compared with the negative control group and the blank control group. HUVEC, human umbilical vein endothelial cells.

**Figure 5:**
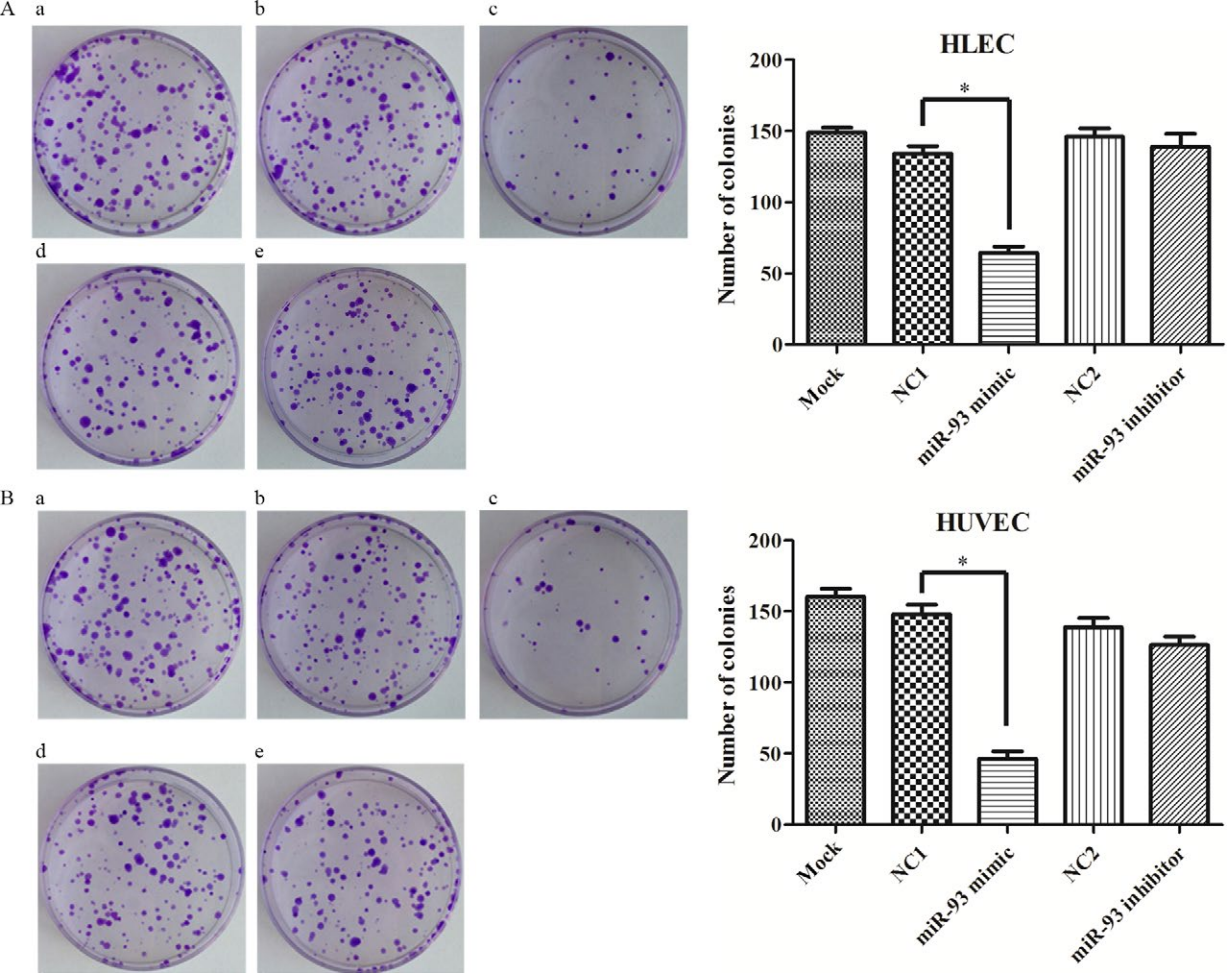
Effect of miR‐93 on cell proliferation in HLEC and HUVEC with clone formation assay. A statistically significant reduction in the number of colonies was observed in miR93 mimic group. (A) HLEC. (B) HUVEC. (a) blank control. (b) and (d) negative control (NC‐miRNA). (c) miR93 mimic. (e) miR‐93 inhibitor. Scare bar = 100 *μ*m. *P <* *0.05. HUVEC, human umbilical vein endothelial cells.

Cell migration and proliferation assays were performed. The transwell cell migration assay was performed (Fig. 3). Cell proliferation was investigated by using the WST‐1 assay (Fig. 4) and clone formation assay (Fig. 5). The data demonstrated that the overexpression of miR‐93 significantly inhibited the migration and proliferation of HLEC and HUVEC. However, after the transfection with mir‐93 inhibitor, the migration and proliferation capacity of HLEC and HUVEC did not change significantly.

### MiR‐93 reduces tube formation capacity of epithelial cells

**Figure 6:**
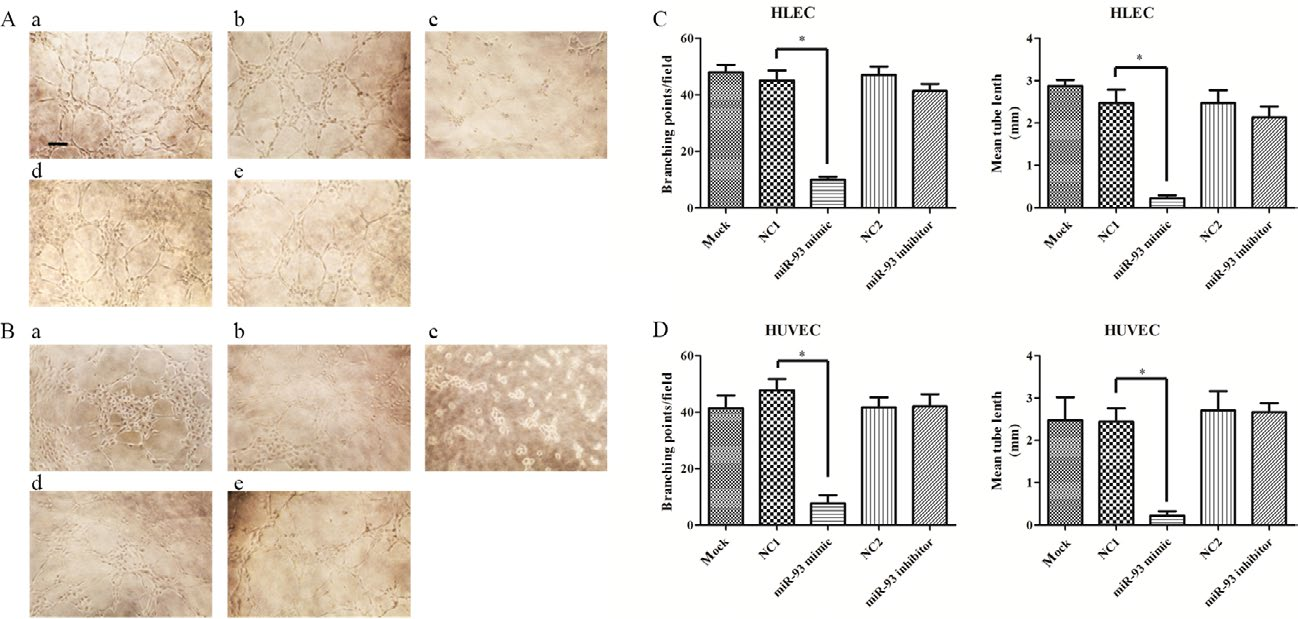
Effect of miR‐93 on cell tube formation capacity in HLEC and HUVEC with Matrigel plug assay. Matrigel assays were performed on HLEC and HUVEC 24 h after the transfection. Results are expressed as number of mean tube length and branching points. Branching points were counted by using MetaMorph. MiR‐93 mimic significantly weakened the tube formation capacity of HLEC (A) and HUVEC (B). Branching points and mean tube length also decreased in MiR‐93 mimic group (C,D). (A) HLEC. (B) HUVEC. (a) blank control. (b) and (d) negative control (NC‐miRNA). (c) miR93 mimic. (e) miR‐93 inhibitor. Scare bar = 50 μm. *P <* *0.05. HUVEC, human umbilical vein endothelial cells.

Angiogenic potential was assessed by Matrigel assays on HLEC and HUVEC in vitro. HLEC and HUVEC were plated on Matrigel coated plates. Compared with blank control and negative control groups, the tube formation capacity of miR‐93 mimic reduced significantly. However, miR‐93 inhibitor group had similar tube formation capacity level with the control group(Fig. 6A and B). Furthermore, the MetaMorph software showed that the length of tube and the number of branching points decreased significantly in HLEC and HUVEC after the transfection with miR‐93 mimic (Fig. 6C and D).

### MiR‐93 regulates apoptosis of epithelial cells

**Figure 7:**
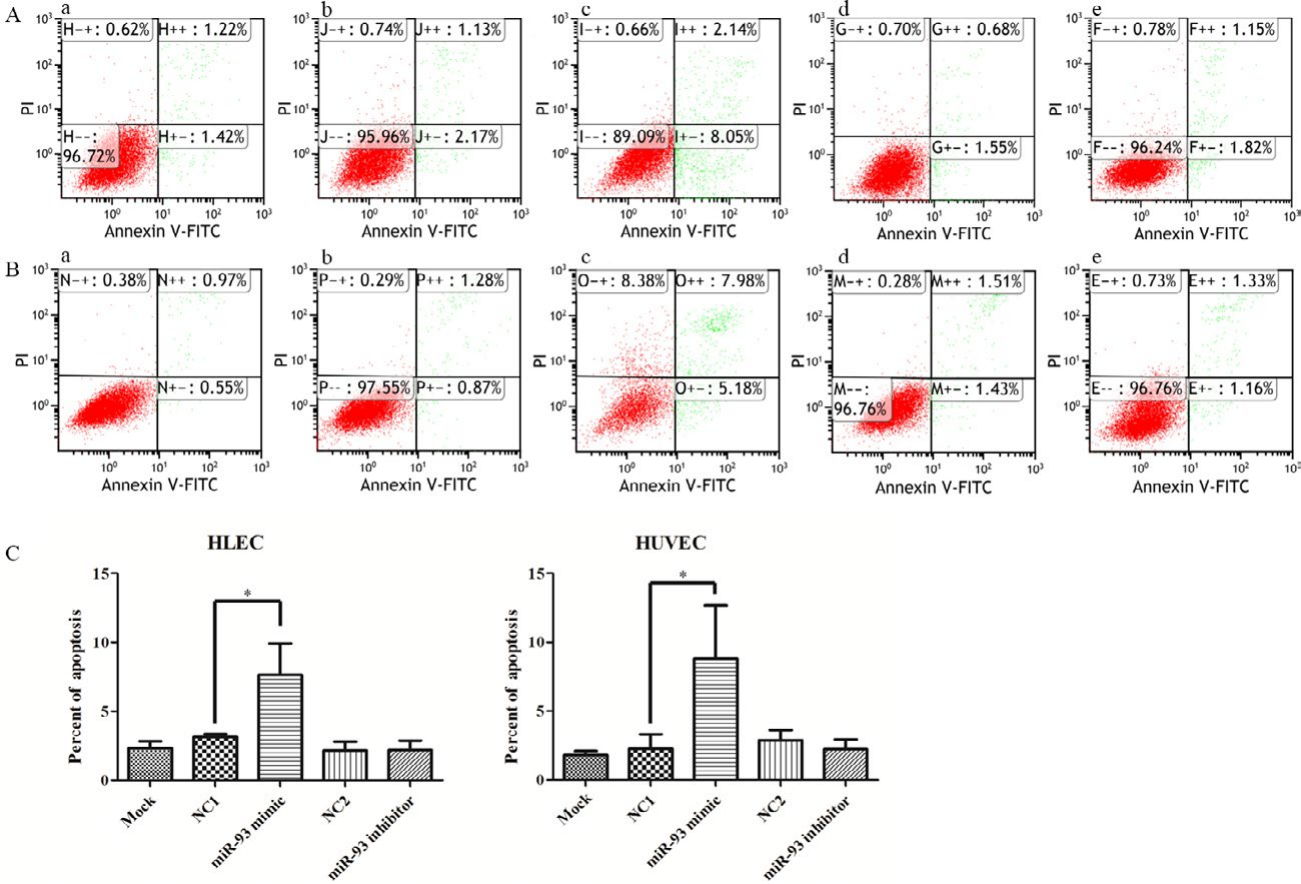
MiR‐93 enhances apoptosis of epithelial cells. A duration of 48 h after the transfection with miRNA mimics, HLEC and HUVEC were collected for apoptosis analysis. The apoptotic rates were detected by flow cytometry (A, B). Results demonstrated the increased apoptosis in miR‐93 mimic group. Results demonstrated the increased percentage of apoptosis cells in miR‐93 mimic group. (A) HLEC. (B) HUVEC. (a) blank control. (b) and (d) NC‐miRNA. (c) miR93 mimic. (e) miR‐93 inhibitor. *P <* *0.05. HUVEC, human umbilical vein endothelial cells. The apoptotic rates were detected by flow cytometry (A, B) and showed in histograms (C).

An annexin V‐fluorescein isothiocyanate (FITC) apoptosis detection kit (Beyotime Biotechnology Company, Jiangsu, China) was used to detect apoptosis. All the samples were assayed in triplicate. Assays were performed on HLEC and HUVEC 48 h after the transfection (Fig. 7). Results demonstrated the increase of apoptosis in miR‐93 mimic group (HLEC: 7.63% compared with blank control 2.32% and NC control 3.13%, HUVEC: 12.88% compared with blank control 1.46% and NC control 1.56%). In miR‐93 inhibitor group, the apoptosis rate of HLEC and HUVEC was similar to the control group, suggesting that this might be related to the high expression of miR‐93 in endothelial cells and the insufficiently functional inhibition 14, 15.

### MiR‐93 changes the cell cycle profile of epithelial cells

**Figure 8:**
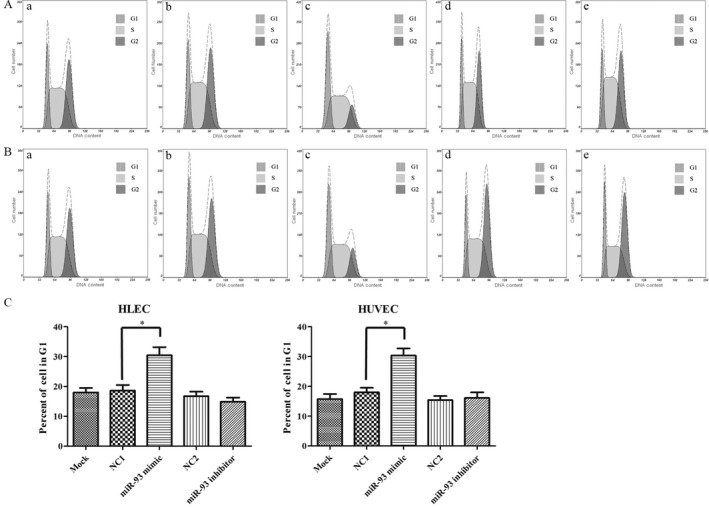
Cell cycle detection after miR‐93 intervention. MiR‐93 changes the cell cycle profile of HLEC and HUVEC. A duration of 48 h after the transfection with miRNA mimics, HLEC and HUVEC were collected and stained with PI. The cell cycle was detected by flow cytometry (A, B). Results demonstrated the increased percentage of G1 cells in miR‐93 mimic group (C). (A) HLEC. (B) HUVEC. (a) blank control. (b) and (d) NC‐miRNA. (c) miR93 mimic. (e) miR‐93 inhibitor. *P <* *0.05. HUVEC, human umbilical vein endothelial cells; PI, propidium iodide.

Two days after the transfection, cells were collected for cell cycle analysis by using propidium iodide (PI) (Beyotime Biotechnology Company, Jiangsu, China) staining assay. In miR‐93 mimic group, flow cytometry results showed a significant increase in the percentage of cells in the G1 peak (HLEC: 30.4% compared with blank control 18.0% and NC control 18.6%, HUVEC: 30.3% compared with blank control 15.7% and NC control 18.0%). In miR‐93 inhibitor transfection group, the percentage of cells in the G1 peak did not decrease significantly, suggesting that this might be related to the high expression of miR‐93 in endothelial cells and the insufficiently functional inhibition Fig. 8C.

### MiR‐93 targets Ang2 3′ UTR

With the help of the online database (link: http://www.microrna.org/microrna/getMrna.do?gene=285&utr=22866&organism=9606), we predicted that miR‐93 can regulate Ang2. The prediction showed that a variety of miRNAs in the family of miR‐17, including miR‐93, miR‐106b, miR‐106a, miR‐20b, and miR‐20a, may have the ability to combine with the 3′ UTR of Ang2. And also, it is confirmed that miR‐93 can regulate Ang2 by acting on the 1933rd of the termination codon of Ang2 mRNA.

**Figure 9:**
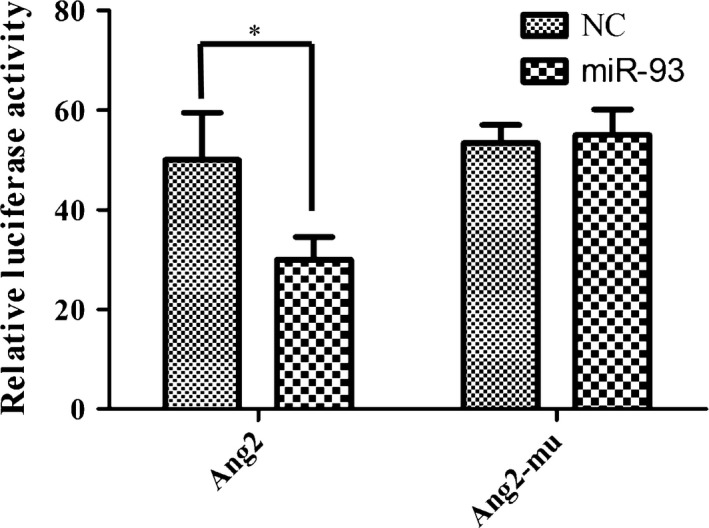
Verification of the binding sites of miR‐93 and Ang2 through luciferase reporter gene experiment. The binding capacity of miR‐93 and 3′ UTR region of Ang2 was detected. Also, specific location of target area was determined through mutation. Ang2‐mu stands for the binding site mutation group; NC stands for the negative control group. *P <* *0.05.

To address whether the binding of miR‐93 and Ang2 3′‐UTR leads to the suppression of Ang2, dual luciferase reporter assay was performed. Also, a mutated miR‐93 binding site plasmid (Ang2 mutant) was generated. The luciferase activity in the miR‐93 mimic group is significantly lower than the NC miRNA group, suggesting that this site was the transcription factor binding site. More importantly, the suppression of miR‐93 mimics on (Fig. 8) Ang2 3′‐UTR was abrogated when cells were transfected with Ang2 mutant plasmid (Fig. 9). Results showed that miR‐93 acts as a negative regulator of Ang2 by binding to the Ang2 3′‐UTR (1933–1956 bp).

**Figure 10:**
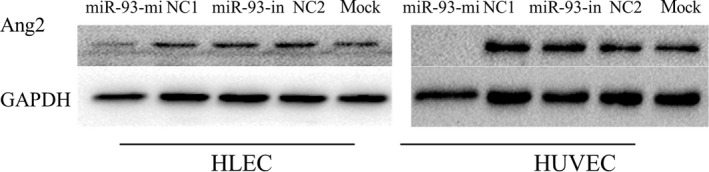
Effect of miR‐93 on the expression of Ang2 protein in HLEC and HUVEC. Protein levels of Ang2 were analyzed by western blotting in whole cell lysates 46 h after the transfection. The expression of Ang2 protein in miR‐93 mimic group markedly decreased. However, the expression of Ang2 protein in miR‐93 inhibitor group showed no increase. Mock: blank control. HLEC, human lymphatic endothelial cells.

By using the monoclonal Ang2 antibody, we further assessed the protein level of Ang2 in epithelial cells, so as to substantiate the connection between miR‐93 and its target(Ang2). Western blot analysis indicated that, compared with the NC control group and the blank control group, the Ang2 protein level significantly decreased after the transfection of HLEC and HUVEC with miR‐93 mimics. However, there is no significant increase in the miR‐93 inhibitor group. This may be related to the incomplete inhibition of miR‐93 caused by the high expression of miR‐93 in epithelial cells (Fig. 10). The results indicate that miR‐93 down‐regulates the Ang2 expression through binding to the 3′ UTR of the Ang2 gene.

## Discussion

Non‐small‐cell lung cancer (NSCLC) is the leading cause of cancer death 16. MPE has been found in 15% of newly diagnosed patients with NSCLC. It is one of the signs of poor prognosis in patients with lung cancer 1. Effectively inhibiting the development of pleural effusion is an important part of the whole treatment. Through the treatment, dyspnea can be alleviated or eliminated, and hospitalization days can be shortened 2, 17. However, active palliative treatment, such as pleural puncture, long‐term placement of drainage tube, and pleural fixation, would lead to high recurrence rates 2, 3. Thus, novel treatment for MPE requires a better understanding of the molecular mechanisms of this disease.

Given the powerful regulatory potential, MiRNAs are ideal therapeutic targets 18. Many studies have identified that miRNAs are associated with MPE 19, 20. Studies on the detection of serum and pleural effusion miRNA showed that the diagnosis or prognosis of diseases is related to a variety of miRNAs 10, 11. In the serum, plasma, pleural effusion, bronchoalveolar lavage, sputum, and saliva of lung cancer patients, distinctive expressions of miRNAs were detected 21. Previous researches showed that the expression difference of miR‐10b can be used as the biomarker to distinguish between benign and malignant pleural effusion 22. Gee GV discovered that the expression level of miR‐200 in malignant pleural mesothelioma effusion is significantly lower than in the pleural effusion caused by lung adenocarcinoma 23. Studies confirmed that the exosome secreted by cells contains miRNA and mRNA. Also, it is discovered that, with some stimulation, cells can selectively wrap miRNA to produce exosomes and send them to the circulatory system. When exosomes, which contain miRNA, enter the target cells, they act as endogenous miRNAs 24, 25, 26, 27. In this study, for the first time, we showed that cell‐free miR‐93 and targeted Ang2 contribute to inhibiting angiogenesis and lymphangiogenesis in the development of MPE.

MiR‐93 belongs to the miR‐17 family. It is in the 13 intron region of mini‐chromosome maintenance protein 7 (MCM7) which is located on chromosome 7q22. Studies found that, in different kinds of tumors, miR‐93 plays different roles, and even leads to some opposite effects. From the intestinal cancer research, we discovered that, miR‐93 inhibited the proliferation and migration through the intervention of genes related to cell cycles, so as to control tumor growth and recurrence 28. Many researches showed its anticancer effects on colon cancer and ovarian cancer 29, 30, 31. It is interesting to find that miR‐93 acts as oncogene in laryngeal cancer, breast cancer, and osteosarcoma 32, 33, 34, 35. From tumor angiogenesis studies, we discovered that, through the co‐culture technique, the overexpressed tumor cells of miR‐93 can promote the proliferation and migration of endothelial cells. Further research showed that miR‐93 may promote the growth of blood vessels by inhibiting the expression of interleukin 8 14, 36. MiR‐93 plays different roles in different tumors or tissues. It may act as oncogenes or antioncogenes. Previous studies have found that the relationship of some miRNAs is somewhat contradictory 37. And also, researches indicated that miRNA can regulate a variety of mRNAs after the transfection. The roles these genes played in different tissues or tumors are different, which may be the reason for the diversity of miRNA functions 38. In this study, by using PCR, we discovered that the miR‐93 expression in MPE of the good prognosis group patients was significantly higher. In the development or the treating course of lung adenocarcinoma with MPE, miR‐93 may inhibit the generation of MPE. However, its target is not clear.

The expression of MiR‐93 can inhibit the migration, proliferation, and angiogenesis of both HLEC and HUVEC cell lines. The results are consistent with the clinical results. As we all know, invasion and metastasis of tumor cells are associated with angiogenesis and lymphangiogenesis, which are also important factors contributing to the formation of pleural effusion 39. However, negative results of relative cell biology experiments (migration, proliferation, angiogenesis assay, cell cycle, and apoptosis) were shown in miR‐93 inhibitor group. Considering the high expression of miR‐93 in endothelial cell lines 14, 15, miR‐93 inhibitor inhibits the expression of miR‐93, but the expression of downstream proteins (for example, Ang2) shows no changes. This may be the reason for negative results. The expression of miR‐93 in the MPE of lung adenocarcinoma patients inhibits the generation of pleural effusion by inhibiting the generation of angiogenesis and lymphangiogenesis. In this way, it suppresses tumor indirectly.

The downstream regulation target of miR‐93 in pleural effusion is not clear. By using database, we discovered that the target gene of miR‐93 might be Ang2. Ang2 is a member of angiogenin family, which was first obtained from the cell culture supernate in 1985. Ang1 and Ang2 participate in vascular regeneration and functional regulation by combining with tyrosine kinase receptor (Tie‐2) on the surface of endothelial cell membrane. Ang1 is capable of anti‐inflammation and anti‐exudation 40. In epithelial cells, Ang2 is antagonistic to Ang1. In this way, Ang2 can increase vascular permeability, and both Ang2 and Ang1 can participate in the occurrence and development of pleural effusion. In the treatment of malignant tumor diseases, Ang2 can regulate vascular stability so as to promote the generation of neovascularization. Besides, Ang2 is related to poor prognosis in non‐small‐cell lung cancer 41. In addition, Ang2 increases significantly in exudative pleural effusion. Ang2 and VEGF participate not only in pleural inflammation, but also in the generation of exudative pleural effusion 42. The latest study confirmed that, by regulating the functional connection of lymphatic endothelial cells, Ang2 plays an important role in the occurrence and development of lymph vessels 11. Clinical studies have found that Ang2 is of great value in the diagnosis, metastasis, and prognosis evaluation of lung cancer and other malignant tumor diseases 5, 40, 41. Further intervention studies show that targeted inhibition of Ang2 can inhibit tumor angiogenesis and lymphangiogenesis. Therefore, Ang2 may be the potential target for anticancer therapy 43, 44, 45, 46. By performing luciferase reporter gene assay and western blot, this study confirmed the regulatory ability of miR‐93 on Ang2 expression on both molecular level and protein level. In miR‐93 mimic transfection group, the expression of Ang2 decreased significantly, while there was no significant increase in miR‐93 inhibitor group. Three independent experiments of this study show that the cycle threshold (CT) values of miR‐93 detected in two cell line blank control group were 28.75 (HLEC) and 27.46 (HUVEC), indicating the high basic expression of miR‐93 in endothelial cell lines, which was confirmed by previous researches 14, 15. In this way, although inhibitor down‐regulates miR‐93 to some extent, the Ang2 expression on protein level will not be significantly affected by the translation.

In summary, by using miRNA microarray and PCR, we made it clear that miR‐93 shows high expression in malignant pleural effusion with good prognosis, which may inhibit pleural effusion. We further confirmed that, with the help of Ang2, miR‐93 may regulate endothelial cell migration, proliferation, angiogenesis, and cell cycle. And also, it may inhibit pleural effusion by regulating angiogenesis and lymphangiongenesis. Research has shown that miR‐93 plays an important role in the occurrence and development of MPE, contributing to developing novel treatments for MPE.

## Conflict of Interest

The authors do not have any conflicts of interest with the content of this manuscript.

## Supporting information


**Table S1.** After normalization of microarray detection results, 107 miRNAs were found to be differentially expressed between good and poor prognosis groups, and 33 of them have fold‐changes ≥2.0.
